# HIV Status Disclosure through Family-Based Intervention Supports Parenting and Child Mental Health in Rwanda

**DOI:** 10.3389/fpubh.2016.00138

**Published:** 2016-06-29

**Authors:** Sumona Chaudhury, Catherine M. Kirk, Charles Ingabire, Sylvere Mukunzi, Beatha Nyirandagijimana, Kalisa Godfrey, Robert T. Brennan, Theresa S. Betancourt

**Affiliations:** ^1^Department of Epidemiology, Harvard T.H. Chan School of Public Health, Boston, MA, USA; ^2^Department of Global Health and Population, Harvard T.H. Chan School of Public Health, Boston, MA, USA; ^3^WE-ACTx for Hope, Kigali, Rwanda; ^4^FXB-Rwanda, Kigali, Rwanda; ^5^Inshuti Mu Buzima, Partners in Health (PIH), Kigali, Rwanda

**Keywords:** children affected by HIV AIDS, Rwanda, family-based intervention, resilience, parenting

## Abstract

**Introduction:**

Few evidence-based interventions exist to support parenting and child mental health during the process of caregiver HIV status disclosure in sub-Saharan Africa. A secondary analysis of a randomized-controlled trial was conducted to examine the role of family-based intervention versus usual social work care (care as usual) in supporting HIV status disclosure within families in Rwanda.

**Method:**

Approximately 40 households were randomized to family-based intervention and 40 households to care as usual. Parenting, family unity, and child mental health during the process of disclosure were studied using quantitative and qualitative research methods.

**Results:**

Many of the families had at least one caregiver who had not disclosed their HIV status at baseline. Immediately post-intervention, children reported lower parenting and family unity scores compared with those in the usual-care group. These changes resolved at 3-month follow-up. Qualitative reports from clinical counselor intervention sessions described supported parenting during disclosure. Overall findings suggest adjustments in parenting, family unity, and trust surrounding the disclosure process.

**Conclusion:**

Family-based intervention may support parenting and promote child mental health during adjustment to caregiver HIV status disclosure. Further investigation is required to examine the role of family-based intervention in supporting parenting and promoting child mental health in HIV status disclosure.

## Introduction

There is a dearth of literature examining the effects of caregiver HIV status disclosure within families in sub-Saharan Africa (1). Notably, there is limited examination of interventions that support parenting and child mental health during this process (2–4). Family-based intervention delivers psychosocial support for children through strengthening communication and parenting, resulting in enhanced disease-coping strategies in both the immediate and long term (5–10). In particular, family-based intervention may support parenting and promote child mental health during the process of caregiver HIV status disclosure to children (9, 10). Early research in South Africa has demonstrated the feasibility of family-based intervention to support caregiver HIV status disclosure to children (11).

Family-based intervention, also known as family strengthening intervention, was adapted for use within HIV-affected families in Rwanda (FSI-HIV) (10–15). Quantitative and qualitative analysis of data from an 80-family randomized-controlled trial of FSI-HIV versus usual-care social work was undertaken to examine supported caregiver HIV status disclosure within families in Rwanda^1^.

## Materials and Methods

Quantitative and qualitative data from a randomized-controlled trial of a family-based intervention (FSI-HIV) versus usual-care social work were examined. Changes in parenting skills, child resilience, and child mental health during the process of supported caregiver HIV status disclosure were assessed (10). Quantitative data were collected at pre-intervention, immediate post-intervention, and at 3 months post-intervention, from December 2012 to June 2014. The relationship between supported HIV status disclosure and family relationships during the family-based intervention was explored through mixed-methods techniques (12–14).

### Study Population

Families affected by caregiver HIV were recruited through referrals from health-center social workers in rural Southern Kayonza District in Rwanda for participation within a randomized-controlled trial of the FSI-HIV. A randomization sequence was generated in Microsoft Excel to assign families to the FSI-HIV intervention or to the control group of the trial. Randomization was conducted after baseline assessments. A sample size of 80 families was calculated, assuming 2 eligible respondents per family on average and moderate intra-class (within-family) correlation (approximately 0.5), to yield power of 0.80 to detect a standardized a “medium” effect size of approximately 0.50 in study outcome measures, assuming a standard alpha level of 0.05. Inclusion criteria required at least one caregiver to be HIV-positive and at least one school-aged child (7–17 years) to be resident within participating households. Caregivers agreed to discuss their HIV status with their children. Caregivers gave informed consent to participate for themselves and for their children. Additionally, children gave oral consent. Children could elect not to participate. A community advisory board was formed to oversee conduct of the study. All study procedures were granted approval by the Rwandan National Ethics Committee and the Harvard School of Public Health’s Institutional Review Board.

### Intervention

The FSI-HIV was designed, developed, and tested within families affected by caregiver HIV in Rwanda (10, 15, see footnote text 1). Previously published findings have demonstrated the acceptability and feasibility of FSI-HIV (10). The four main aims of the FSI-HIV comprise development of resilience through family narrative, improved parenting and family communication, HIV psychoeducation, and engagement of formal and informal sources of support (10, 15). Data about demographics and HIV status of all family members were collected in introductory meetings. Then, trained bachelor-level counselors delivered a series of six core modules within each household. Counselor-led sessions with caregivers (Modules 1, 2, and 4) established the family narrative, discussed the effect of HIV on the family, and identified sources of resilience. Counselor-led sessions with children (Modules 3 and 5) established the family narrative from the children’s perspective, provided psychoeducation on HIV, and identified sources of resilience. During the sixth and final module, caregivers led a family meeting and discussed the family’s challenges, strengths, and goals (10).

### Controls

Once enrolled in the study, participating households were randomized to receive the FSI-HIV intervention or care as usual social work. Control households received care as usual social work support through the locally available government-provided social services. This support generally consisted of advice concerning food insecurity and access to schooling. Study outcomes were measured in both FSI-HIV and control households at baseline, immediately post-intervention, and at 3-month follow-up.

### Study Outcomes

#### Parenting and Family Unity

Parenting was measured using local and combined parenting scales consisting of a 32-item scale (α = 0.91). The local parenting score contained 16 locally derived items (4), whereas the combined parenting score included an additional 16 items from the Parental Acceptance and Rejection Questionnaire, scored on four-point scale from 0 (“never”) to 4 (“every day”) (16). Family unity was assessed on a scale of 0 (never) to 3 (every day), using a 15-item scale derived from local qualitative data (α = 0.93) (4, 10). Parenting and family unity scores were developed and validated within Rwandan families in prior mixed-methods studies (15). Questionnaire components capture varying dimensions of parent–child and family relationships (Table 1) (15).

**Table 1:** **Components of the parenting and family connectedness assessments**.

Good parenting	Family connectedness
Provide trainings	Interact with each other
Provide teachings	Converse to reach agreements
Provide discipline	Understand each other
Give advice	Unified
Converse with children	Do not have conflicts with each other
Interact with children	Being honest with each other
Draw close to children	Not suspicious of each other
Treat all children in the family equally	Cooperate with each other
Respect children	Respect each other
Being calm with children	Do not stigmatize one another
Express love	Love each other
Provide resources (food, water, clean clothes and school fees)	Share and keep secrets with each other Parents don’t cheat on each other

#### Child Mental Health

Child depression was measured using a locally validated version of the Center for Epidemiological Studies Depression Scale for Children (CES-DC) (17). Child combined anxiety–depression was measured using a 23-item adapted youth self-report (α = 0.93) scored as the mean of items from 0 (“not at all true”) to 3 (“often true”) (18). Child resilience was measured using an adapted Version of the Connor–Davidson Resilience Scale (CD-RISC) (19) and from local qualitative data (α = 0.92) and scored as the sum of all items. Child prosocial behavior was measured using a 20-item scale from local qualitative data (α = 0.90) scored as the mean (4).

#### Data Collection

Quantitative child and caregiver self-report measures of family factors and child mental health were developed and adapted to fit the local context and underwent forward and back translation processes (4, 20). Questionnaires were administered by local research assistants in Kinyarwanda using hand-held smartphones at baseline, immediately post-intervention, and at 3-month follow-up. Qualitative data were extracted from counselors’ clinical notes to capture interventionist observations during child, caregiver, and family interviews through the course of the modules of the intervention.

#### Data Analysis

Quantitative analyses were performed using STATA 13.0. Means of child and caregiver self-reported parenting and child resilience and mental health scores with corresponding 95% confidence intervals were calculated and plotted.

Qualitative data were analyzed using thematic content analysis to identify and analyze patterns driven by *a priori* research questions (21): (1) What, if any, are the effects of the process of HIV disclosure within FSI-HIV families on the relationship between parents and their children? (2) What, if any, are the effects of the FSI-HIV intervention on the process of HIV disclosure with respect to parenting skills and child resilience and mental health? Data were analyzed inductively to identify codes, which were then further categorized to capture main patterns within the data. Themes from families’ experiences were observed and developed from these categories.

## Results

### Baseline Characteristics

Forty-one families were randomized to the FSI-HIV intervention and 41 families to treatment as usual. Approximately half of all families were dual-caregiver households. Most caregivers within FSI-HIV families were female (*n* = 42; 68.9%), HIV-positive (*n* = 52; 85.3%), and had a mean age of 41 years. The majority of children within FSI-HIV families attended school (*n* = 87, 96.7%) and 6.5% were HIV-positive (*n* = 6) (see Table 2).

**Table 2:** **Baseline characteristics of participants enrolled in family-based preventive intervention arm of trial**.

	FSI-HIV	TAU (usual-care control families)
**Families, no. (%)**	41 (50)	41 (50)
Dual-caregiver families, no. (%)	20 (48.8)	20 (48.8)
Average no people per household, mean (SD)	5.1 (1.5)	4.8 (1.5)
Average no children per household, mean (SD)	3.2 (1.3)	3.0 (1.4)
SES, mean (SD)	0.11 (0.08)	0.10 (0.07)
**Caregivers, no. (%)**	61 (49.6)	62 (50.4)
Female, no. (%)	42 (68.9)	42 (67.7)
Age, mean (SD)	41.1 (9.1)	41.0 (8.5)
HIV-positive, no. (%)	52 (85.3)	51 (82.3)
**Children, no. (%)**	93 (54.7)	77 (45.3)
Female, no. (%)	52 (55.9)	31 (40.3)
Age, mean (SD)	11.8 (2.8)	11.7 (2.9)
Attends school, no. (%)	87 (96.7)	64 (88.9)
HIV-positive, no. (%)	6 (6.5)	15 (19.5)
**Non-disclosure families, no. (% of all)**	18 (43.9)	
Maternal, no. (%)	10 (55.6)	–
Combined maternal and paternal, no. (%)	7 (38.9)	–
Paternal, no. (%)	1 (5.6)	–
**Prior disclosure families (% of all)**	17 (41.5)	–
Maternal, no. (%)	13 (76.4)	–
Combined maternal and paternal, no. (%)	4 (23.5)	–
Paternal, no. (%)	2 (11.8)	–
**Supported disclosure families, no. (% of all)**	15 (36.7)	–

### Caregiver HIV Status Disclosure

A total of 18 (43.9%) of all FSI-HIV families experienced non-disclosure of at least 1 caregiver at baseline (see Table 2). The majority of families described maternal HIV status non-disclosure, with a lesser proportion of combined maternal–paternal HIV status non-disclosure and a minority of paternal HIV status non-disclosure. Within these families with non-disclosed HIV status at baseline, 15 went through a supported disclosure process (83.3%) representing approximately 37% of all families enrolled in the FSI-HIV arm of the trial. A total of 17 further families had experienced disclosure of caregiver HIV status in the past, which again had been predominantly maternal HIV status and combined maternal–paternal HIV status disclosure events. A small number of families described unstable, partial, or presumed disclosure experiences.

**Figure 1:**
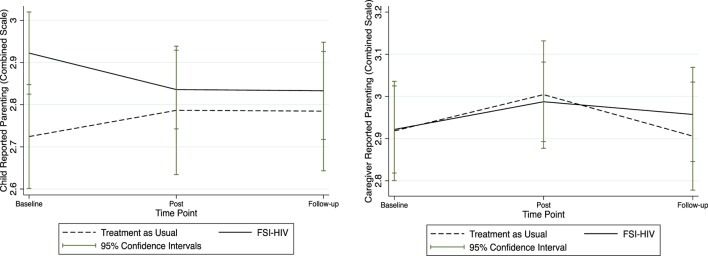
**Child and caregiver mean self-reported parenting scores**. Mean child and caregiver self-reported parenting scores at baseline (pre-randomization), immediately post-intervention, and at 3-month follow-up.

**Figure 2:**
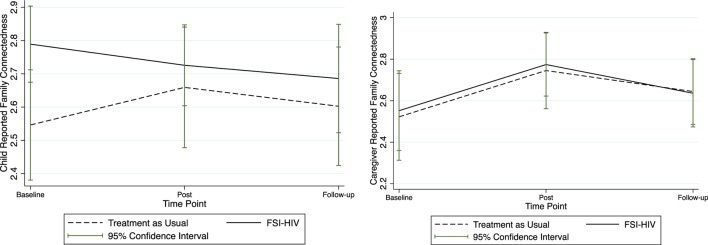
**Child and caregiver mean family unity scores**. Mean child and caregiver self-reported family unity scores at baseline (pre-randomization), immediately post-intervention, and at 3-month follow-up.

**Figure 3:**
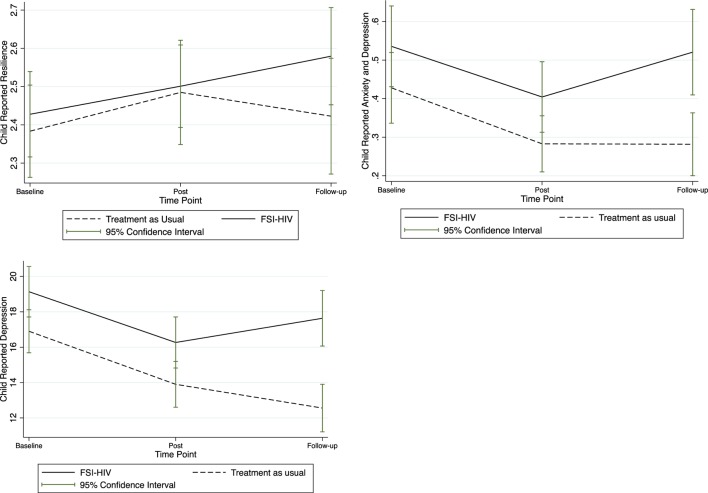
**Child mean self-reported resilience and mental health**. Mean child self-reported resilience, anxiety, and depression, and depression scores at baseline (pre-randomization), immediately post-intervention, and at 3-month follow-up.

Mean scores of all study outcomes were graphically displayed with corresponding confidence intervals over the pre-intervention, post-intervention, and at 3-month follow-up after for FSI-HIV intervention and control families (Figures 1–3).

### Parenting and Family Unity

#### Quantitative

Trends in parenting are displayed through graphical representation of mean self-report scores and corresponding 95% confidence intervals in Figure 1. Child-reported mean parenting scores appear to decrease post-intervention among the FSI-HIV participants compared with apparent increases in the treatment-as-usual arm of the trial, while caregiver-reported parenting scores appeared to increase post-intervention (Figure 1). Both local and combined mean parenting scores remained stable in the FSI-HIV arm of the RCT by 3-month follow-up. Patterns in parenting score reports were closely mirrored by changes in family connectedness scores, reflecting commonalities within the measures concerning communication and trust (Figures 1 and 2).

#### Qualitative

Caregiver HIV diagnosis and disclosure were noted to have effects on the family through a number of potential mechanisms. Caregivers reported reduced coping at the time of HIV diagnosis and disclosure. Grieving processes undermined caregiver capacity to care for children. Additionally, caregivers reported increased marital stress and conflict. Children underwent adjustment responses during the disclosure process. Parental self-assessment of parenting improved during the intervention. Child assessment of parenting also improved, following an initial period of adjustment following caregiver HIV status disclosure.

Since being informed they are HIV positive, they have lost hope and cannot work because they think they will die tomorrow. They lose confidence in their partner or the entire family, and progressively become depressed.

The parents accuse each other and the children think that their parents are going to die and worry about becoming orphans. The children have dropped out of school and are not happy at home as they only see their parents arguing. As the parents are not coping well, everyone in the family is affected.

First parents have to have hope themselves without hopelessness because when children see their parents planning and caring for them, they automatically have hope. If parents have a good relationship, HIV would not be a problem for their children otherwise they think that their parents will die soon. (Counselor, Module 2)

Children described stress responses to parental HIV diagnosis and disclosure. Frequently children described assuming caregiving responsibilities for the family in response to family stress.

When she (the mother) was informed of her HIV serostatus, she became sick. Her first-born tried to help her even though he was very young. He was asking her what he could prepare for her, and he did his best to organize the house and to comfort his young brother and sister. The Holy Spirit motivated him to do so until her mother got some strength.

Overall life in the family changed, the children were depressed and had to work and look after their mother. The elder sister stopped schooling in order to take care of her mother. (Counselor, Module 2)

Partial or unstable parental disclosure was associated with worse effects on child mental health when compared with full disclosure. Children feared discovering the diagnosis from outside of the family and possible community stigmatization. This undermined trust in caregivers contributing to reduced child-reported parenting scores.

Everything is changing, children are losing trust in their parents and are looking for comfort elsewhere. It would help them to know the diagnosis from the parents and not hear it outside and also children would feel trusted. (Counselor, Module 2)

Family was arguing, quarreling, because there was no proper channel of communication and the result was poor family functioning and poor school performance for children. (Counselor, Module 2)

Family intervention offered structured support for improved parental child communication with improvements in family relationships and child mental health. The FSI-HIV provided support to parenting.

The caregiver did a great job in leading the session especially in HIV discussion. It was constructive to her children and she gave them a comforting message. The caregiver mentioned that the discussion was helpful to discuss HIV with the child and the child would ask how her mom became HIV positive, and how she can herself be prevented from HIV/AIDS. (Counselor, Module 4)

I learnt different things about HIV and I was very happy. I wish to continue the conversations. (Final Family Meeting, Child 11 years)

### Child Resilience and Mental Health

#### Quantitative

**Figure 4:**
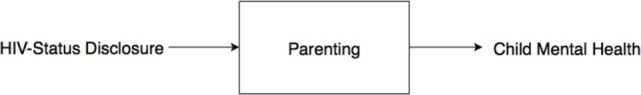
**Directed acyclic graph: mediation of effect of HIV status disclosure on child mental health *via* parenting**.

Child-reported resilience and mental health quantitative scores increased over the course of the intervention as displayed in Figure 3. Further investigation of the possible mediating role of the intervention in improving child mental health through supported parenting in HIV-affected families undertaking disclosure is warranted (Figure 4).

#### Qualitative

Child resilience and mental health improved during the process of disclosure within intervention families.

HIV status is now no longer a major problem for the family. Interventionist assured the mother, that through parenting skills and communication she will be empowered and can learn which proper channels to use in order to handle those issues. (Counselor regarding Mother, Module 2).

I learned how to tell my children about my status; I feel very relaxed about it and I hope that I will be able to do it in this week. Before when I thought about it, it was like a heavy burden and very difficult but now I think that is easier. (Counselor describing Mother’s response, Module 2).

The family group sessions went well, I enjoyed being part of the family group sessions. We discussed about many things including child behavior, HIV/AIDS and how to talk to children and understand them, and be there for them in order to have time for talk. After the family meeting led by FSI-HIV interventionist we would like to go on by holding regular family meetings in order to avoid depression and not feel down. (Counselor describing Mother, Family Review)

Resilient caregivers can make their children resilient. (Counselor, Family Review)

Children described improvements in communication, trust, and honesty. Additionally, children reported greater hopefulness, resilience, and confidence in the family over the course of the intervention.

She said that he enjoyed the session because she learned more about HIV while the caregiver was satisfied because before it was hard for her to discuss with her children about HIV but then after the family meeting she felt relaxed. She was happy also because her children didn’t have emotional problems while talking about HIV in the family. The family is very happy, children did well in school and the whole family is proud of it! (Follow-up Family Meeting)

## Discussion

Disclosure of caregiver HIV status to children can be challenging and is often an ongoing process rather than a one-time event. Disclosure within a family environment is important to facilitate communication about HIV between family members (22). However, caregivers often fear negative consequences of disclosure such as being stigmatized or causing distress to their children (23). However, evidence suggests that caregiver HIV status disclosure conveys psychosocial and clinical benefits for HIV-affected families (24, 25).

### Parenting

At the end of the FSI-HIV intervention, family unity and child-reported parenting appeared to decrease in intervention families, with recovery and signs of resolution after the follow-up period (Figures 1 and 2). Trust was a common component to both the family unity and parenting scale scores. This suggests the possible mechanism of disrupted trust between parents and children underlying reduced parenting and family unity scores immediately surrounding the disclosure process. Discordance between caregiver and child-reported parenting may indicate differences between child and caregiver perspectives of relationships during family-based intervention and support of the disclosure process. In particular, while parents were gaining confidence in their parenting skills through the counseling sessions, children were adjusting to the disclosure of their caregivers’ HIV status. Supported parenting at this time contributed to improved family trust and unity and enhanced child resilience and mental health.

Increasing numbers of children are being affected by caregiver HIV, due to expansion of ARV programs across sub-Saharan Africa. The family is an important focus for intervention for delivering psychosocial support including child protection (6, 11, 26, 27). Prior studies have investigated the impacts of the mediating role of parenting in reducing the impact of caregiver distress on child well-being in HIV-affected families (28). Stress on children surrounding the process of HIV status disclosure may be mitigated through supporting parenting *via* family-based intervention (28). Parenting competence is defined by caregivers’ self-efficacy through self-estimation of competence or ability to positively influence the development of their children in their parenting role (29). Caregivers with psychological distress lose self-esteem as caregivers or may perceive they lack knowledge and skills to provide a suitable environment in which to care for their children (30, 31). HIV-positive mothers’ major concern is their perceived inability to provide adequate care to their children when they became ill (32). Parenting behaviors, such as the maintenance of daily routines, may protect children when a parent is infected with HIV (33). Hence, supporting the parental role, through recognition of its centrality within the family and empowerment of caregivers with a sense of self-efficacy, may be of critical value during HIV status disclosure. Further assisting with parental competences and parental stress management through family-based intervention could contribute toward positive parental coping and reduction of harsh parenting (34, 35). Therefore, it is likely that improvement of parenting protects the mental health of children within the family during caregiver HIV status disclosure.

### Child Mental Health

Children in HIV-affected families living in situations of compound adversity are more frequently called upon to assume adult roles in response to diminished capacity of caregivers to assume responsibilities. This shift in roles is thought to contribute to a negative series of effects on child mental health (36, 37). Burdens on child mental health are exacerbated in situations where there is partial disclosure with a consequent lack of trust within-family relationships and fear of community stigmatization (37, 38).

Child resilience and mental health were shown to improve over the course of the intervention in FSI-HIV families, when compared with control families (Figure 3). The causal directed acyclic graph (DAG) in Figure 4 maps assumptions about potential causal relationships between HIV status disclosure, parenting, and child mental health (39). The effects of HIV status disclosure on child mental health during the trial were potentially mediated *via* parenting, as delineated in the DAG (Figure 4). Hence, by stabilizing changes in parenting following disclosure through family-based intervention, the potential harmful effects of caregiver HIV status disclosure on child mental health were mitigated. Improvements in child mental health within intervention families were also potentially mediated *via* improvements in parenting (40, 41).

### Limitations

Counselors were not blinded as to whether they were offering the family-based intervention or usual-care social work. Baseline measures were undertaken prior to randomization. Hence, apparent differences at baseline between intervention and control groups in Figures 1–3 are artifacts of the randomization process. There was insufficient evidence to justify repeating the randomization, which is generally reserved for extreme situations. There was insufficient justification to select variables for blocking (other than single- versus dual-caregiver status) prior to randomization. Qualitative data from control families were not available; hence, it was beyond the scope of this study to qualitatively compare control families’ experiences of disclosure (42).

## Conclusion

Culturally appropriate interventions are urgently called for to better support parenting during caregiver HIV status disclosure to protect and promote child mental health. Multidimensional assessments are needed when developing and testing interventions for HIV-affected families, to evaluate parenting and family trust during the disclosure process. Future longitudinal studies are called for, to discern the effects of family-based intervention on parenting and child mental health within families undertaking caregiver HIV status disclosure. Further investigation may also illuminate potential mediation of the effect of caregiver HIV status disclosure on child mental health *via* parenting.

## Author Contributions

SC: conception or design of the work, interpretation of data for the work, drafting the work, and agreement to be accountable for all aspects of the work in ensuring that questions related to the accuracy or integrity of any part of the work are appropriately investigated and resolved. CK: contributions to conception and design of work, revising and drafting the work for important intellectual content approval of the version to be published. CI: revising and drafting the work for important intellectual content. SM, BN, KG, and RB: interpretation of data for the work. TB: contributions to conception and design of work, revising work critically for important intellectual content, final approval of the version to be published, and agreement to be accountable for all aspects of the work in ensuring that questions related to the accuracy or integrity of any part of the work are appropriately investigated and resolved.

## Conflict of Interest Statement

The authors declare that the research was conducted in the absence of any commercial or financial relationships that could be construed as a potential conflict of interest.

## Footnotes

^1^Betancourt TS, Ng L, Kirk C, Brennan RT, Beardslee W, Stulac S, et al. Family-based promotion of mental health in children affected by HIV (2016, submitted).
